# Effects of rhythmic auditory guide on sprint running

**DOI:** 10.1371/journal.pone.0319738

**Published:** 2025-03-21

**Authors:** Shinnosuke Hase, Kento Nakagawa, Shigeo Iso

**Affiliations:** 1 Graduate School of Sport Sciences, Waseda University, Saitama, Japan; 2 Faculty of Sport Sciences, Waseda University, Saitama, Japan; Opole University of Technology: Politechnika Opolska, POLAND

## Abstract

Auditory guides can influence the tempo of rhythmic movements, potentially enhancing running performance by improving parameters. Many previous studies have focused on low-speed running, but there is a lack of research on high-speed sprint running. This study investigated whether a metronome-based rhythmic auditory guide could modulate sprint running motions. Twenty-two junior high school students participated in the study, performing three 40-meter sprint trials under different conditions: (1) without an auditory guide (baseline), (2) with a slow-rhythm auditory guide (3.40 ±  0.24 Hz), and (3) with a fast-rhythm auditory guide (4.16 ±  0.29 Hz). Step rate, step length, and sprint velocity were analyzed using video recordings. The slow-rhythm auditory guide significantly decreased the step rate and increased the step length compared with the baseline condition (p <  0.05). Conversely, the fast-rhythm auditory guide significantly increased step rate and decreased step length (p <  0.05). These findings demonstrate that rhythmic auditory guides can effectively alter step rate and step length during sprint running in junior high school athletes. This suggests that auditory stimulus training could be a useful tool for coaching.

## Introduction

In sports coaching, previous studies have reported that a coach’s verbal cues and instructions influence specific aspects of sprint running performance [1–3] such as running time [1, 2]. The use of verbal cues and instructions does not necessarily improve sprint performance for all runners. A previous study that examined the performance effects of instruction on teaching in track and field for male and female students across a wide range of skill levels reported that instruction was effective only for boys and students with higher skill levels, with no significant performance improvement observed in other students [4]. This finding suggests that the coach-directed approach can be influenced by the gender and skill level of the student in terms of performance effectiveness [4]. Therefore, nonverbal methods (e.g., music or metronome) are critical to address the limitations of instructor-based coaching in guiding athletes’ movements.

Running involves a cyclic movement that alternates between the supporting phase (foot landing on the ground) and the recovery phase (foot taking off from the ground) [5]. Performance effects in cyclic movements, known as sensorimotor synchronization, have been observed using periodic tempo and have been the subject of numerous studies [6–9]. Exercising with sound has been reported to increase exercise duration [10], alleviate fatigue [11], and improve running performance [9,12]. Synchronization of movement and sound is often examined using an experimental model of low-to-moderate-intensity exercise [11–13]. Rhythmic sound guidance allows accurate anticipation of subsequent movements, potentially reducing the metabolic cost through improved neuromuscular coordination [12,14]. For instance, previous studies on low-velocity or long-distance running have reported that using an auditory guide (e.g., metronome, music) with a faster tempo than the participant’s natural running step rate increases step rate, while using a slower tempo auditory guide decreases step rate [15, 16]. A study comparing running time to exhaustion on a treadmill with and without a metronome at the same tempo showed a significant increase in running time with the metronome (without metronome: 624 s, metronome: 746 s), suggesting that an auditory guide may delay fatigue in long-distance running (running velocity range: 9.5–17.5 km/h) [9]. Sprint running is important to maintain velocity because of fatigue in the middle and last part of the race [17]. Therefore, even in sprint running, the intervention of the rhythmic sound is expected to contribute to accelerating in the first half and preventing the decrease in the running velocity in the latter half of the race.

High-level sprint runners have been observed to synchronize their movement tempo with external stimuli, such as the step rates of adjacent competitors. Varlet and collaborators conducted a race analysis of 100m races at the World Championships, measuring the step rate of each athlete’s run [18]. They revealed that the participants’ step rate was synchronized with the step rate of athletes in adjacent lanes, indicating that synchronization occurs even in high-velocity sprint running. Therefore, it is conceivable that synchronization can occur in high-velocity sprint running as studies of low-velocity running [9,15,16].

However, several previous studies have primarily focused on low-velocity running [9,15,16], and the potential to modify high-velocity sprint running movements through an auditory guide remains unclear. Furthermore, many synchronization studies using auditory guides and running have been conducted in laboratory settings on treadmills [9,16,19], with limited experiments conducted outdoors on actual running fields [12,15].

Based on these observations, we hypothesized that the step rate in high-velocity sprint running can be modified by synchronizing the motion with a rhythmic auditory guide. If the current study supports our hypothesis, this approach could be implemented by tailoring sound tempo adjustments to target specific spatiotemporal parameters, such as step rate or length, for individual athletes. Thus, in this study, we aimed to investigate the effects of auditory guidance on the spatiotemporal variables of high-velocity sprint running in junior high school students.

## Materials and methods

### Participants

In this study, the participants were required to perform sprint running. Therefore, we recruited students from junior high school track and field clubs who were accustomed to sprint running. The club adviser was asked to recruit participants, and they subsequently sent an email to all club members informing them of the study. There were no restrictions on age or event, as long as the participants were junior high school students belonging to the club. Students who were injured and unable to perform their full performance were excluded. To establish the minimum sample size, power analysis for a repeated-measures design was conducted using G * Power 3.1.9.7 [20]. Using an effect size, alpha, and power of 0.4, 0.05, and 0.95 a minimum of 19 participants were required. We recruited a total of 22 participants for this study. This study included 22 junior high school students (boys = 11, girls = 11) who were members of a track and field club. The group consisted of 18 sprint runners and 4 long-distance runners. Part of all the students’ training included sprint running 2–3 times a week; therefore, the students were accustomed to performing sprint running. The mean age was 13.18 ±  0.65 years, the mean height was 1.60 ±  0.06 m, and the mean weight was 48.73 ±  4.46 kg. The personal best average in the 100 m was 13.86 ±  1.40 s in sprint runners (n = 18). The personal best average in the 800 m was 2 min 47.03 ±  15.08 s in long-distance runners (n = 3). One of the long-distance runners had never previously run an 800 m race (n = 1).

Informed consent was obtained from the students, their parents, and club advisers before the experiment. The study’s background, purpose, methods, consent to participate, and handling of personal information were explained both in writing and verbally to the students and the club advisers in the presence of the investigator Subsequently, students’ parents were provided with written documents containing the same information. Written informed consent, which included signatures from both parents and students, was obtained if both agreed to participate. This study was approved by the Ethics Review Committee on Research with Human Subjects of Waseda University (2022-115).

### Tasks

The participants performed a warm-up exercise routinely in track and field clubs before the experiment. The warm-up exercise lasted for 30 min and prepared the participants to run with submaximal effort. The experiment consisted of three 40 m submaximal sprint running tasks: 1) sprint running without a metronome (baseline), 2) sprint running with a slow-rhythm metronome auditory guide (slow-rhythm), and 3) sprint running with a fast-rhythm metronome auditory guide (fast-rhythm). For the slow- and fast-rhythm tasks, we set up the metronome frequency to ± 10% of the step rate based on the initial 5 m run during the baseline task for each participant (slow-rhythm: 90% of the step rate at baseline; fast-rhythm: 110% of the step rate at baseline) [21]. The average metronome frequency was 3.40 ±  0.24 Hz for the slow-rhythm task and 4.16 ±  0.29 Hz for the fast-rhythm task. The metronome frequencies based on video footage were calculated using the first task, derived from the baseline data. Subsequently, slow- and fast-rhythm tasks were conducted randomly, with a 10-min rest interval between trials.

For sprint running intensity, we instructed the participants to run with a subjective effort of 80% based on their maximum effort during the baseline preliminary experiment [22, 23]. The participants were instructed to “run at what you perceive to be 80% of your full velocity for the baseline task.” In the slow- and fast-rhythm tasks, the participants were instructed to “run in synchronization with the metronome.” We assessed their ability to synchronize their sprint running with the auditory guide, not specifying when they should synchronize with the metronome auditory guide (e.g., during ground contact or arm swing). Therefore, participants individually synchronized their running with the sounds using their synchronization methods. All tasks started from a “standing start position,” participants were asked to run past the 45 m mark to prevent deceleration before reaching the goal.

### Materials

**Fig 1:**
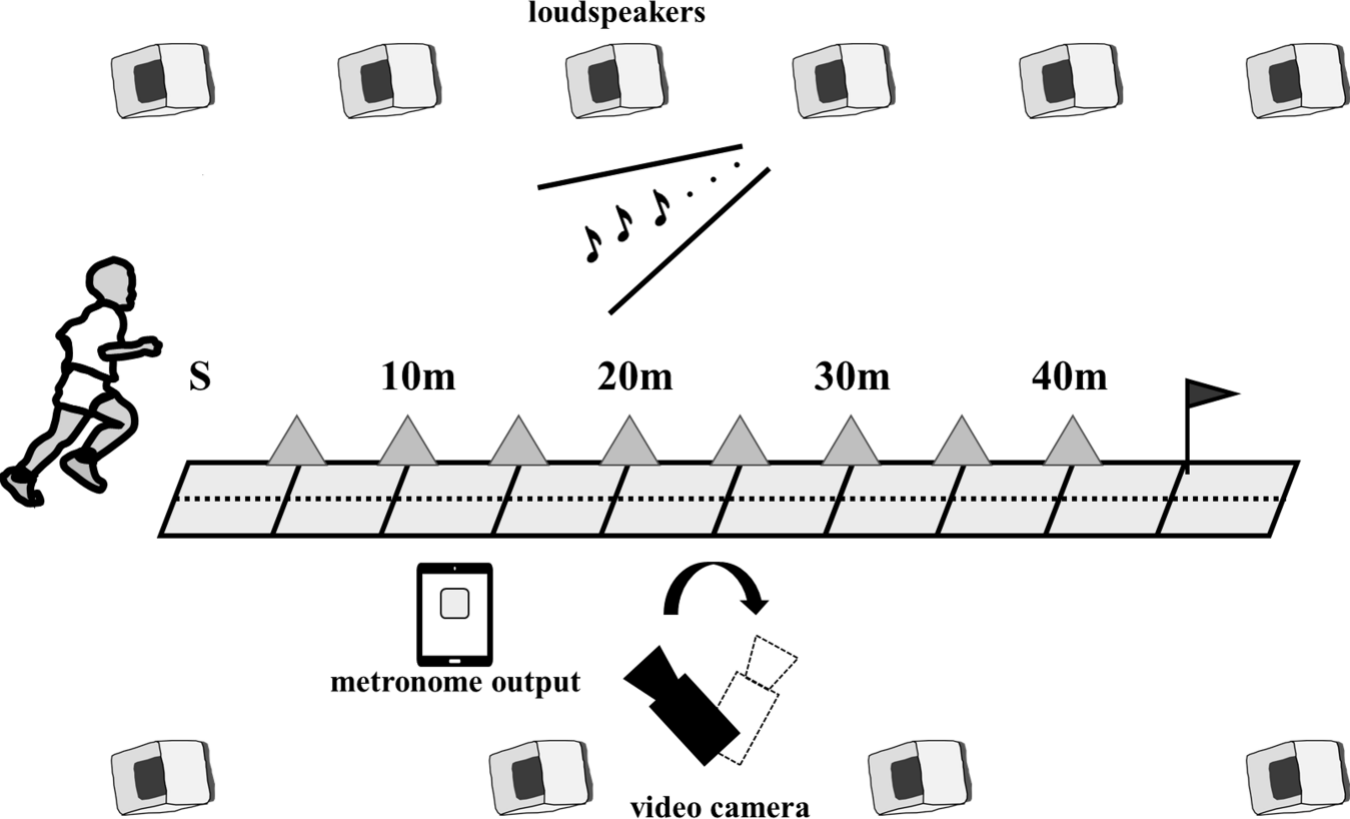
Experimental setup.

This study was conducted in a gymnasium to exclude the effects of wind on performance. The gymnasium, measuring 34 m x 44 m, was equipped with eight speakers (AMU208, BOSE, MA, USA) and two subwoofers (MB210-WR, BOSE). Participants sprinted diagonally across the gymnasium. A digital video camera (frame rate: 240 frames/s; DMC-FZ300, Panasonic, Osaka, Japan) was placed beside the 20 m mark and panned with a tripod to film the running movement from the start to the 40 m mark. A light-metronome device (iPad Air 4th generation, Apple, California, USA) was placed 15 m from the side and was captured by the video camera. The light-metronome device could output the screen at 30 Hz. In this study, video was recorded using a camera with a frame rate of 240 Hz, which clearly captured the moments when the light-metronome device emitted light. Therefore, the videos recorded when the foot touched the ground and when the sound was emitted. The metronome was connected to the light-metronome output device, and an analog mixer (WR-X22, Panasonic) was installed in the gymnasium to broadcast the sound through the speakers. We used an application that produces sound and light simultaneously (The Metronome by Soundbrenner, Soundbrenner Inc., Berlin, Germany). Markers were placed every 5 m along the track to calibrate a digital video camera (Fig 1). The participants could hear the sound of their footsteps and the metronome through the speakers. However, the introspection reports of the participants after the trial indicated that they were primarily aware of metronome sounds.

### Measures

We calculated the sprint velocity, step rate, step length, stance time, and flight time for each 5 m interval from the start to the 40 m mark [24]. We defined the start as when the back leg left the ground. The time from the start to 5 m was defined as the time the back leg left the ground until the torso (chest and abdomen) crossed the 5 m marker cone. We calculated the time for each 5 m interval from the 5 m to 40 m mark as the period from when the torso crosses one marker cone to when it crosses the next. The step rate was calculated by dividing one cycle (two steps), defined as the period when the first foot touched the ground within 5 m, by the time required for that cycle. Because sprint velocity is the product of step length and step rate, we calculated step length by dividing sprint velocity by step rate. Stance time was defined as the period from when the toe touched the ground to when it left. Flight time was defined as the period when neither foot touched the ground, which is the period between the toe leaving the ground and when it touched the ground again. Stance and flight times were averaged over one cycle (two steps). For participants who touched the ground for more than one cycle within a 5 m interval, the average stance and flight times of the first cycle were used.

We evaluated the synchronization between sprint running and metronome sounds by calculating the relative phase between the moment of metronome output and the moment the toe touched the ground. The formula proposed by Varlet et al. [18] was used as a reference.


$$\begin{array}{c} Relativephase\left({}^{\circ}\right)=\frac{T_{td\left(n\right)}-T_{mn\left(n\right)}}{T_{mn\left(n+1\right)}-T_{mn\left(n\right)}}\times 360 \end{array}$$ (1)

$T_{td}$: Time from start to the moment of touching the ground

$T_{mn}$: Time from start to the moment sound (light)

$T_{mn\left(n+1\right)}-T_{mn\left(n\right)}$: Required time from the moment sound (light) to the next sound (light)

The relative phase for each step was calculated from the initial time up to the 40 m mark. The relative phase corresponds to the timing of the first touchdown and first metronome sound from the start. We normalized the relative phase from ‒180° to 180° as in previous studies [18]. A positive relative phase indicates that the moment of touchdown was later than that of the metronome, whereas a negative phase indicates that touchdown occurred earlier. We defined a relative phase of 0° to ± 90° as “in-phase” and a relative phase of 90° to 180° and ‒90° to ‒180° as “anti-phase.”

### Statistical analyses

A two-way repeated-measures analysis of variance with a Greenhouse-Geisser correction was used to test each measurement (3 tasks ×  8 intervals of 5 m from the start to the 40 m mark), and post-hoc analyses were conducted using the Bonferroni method when a main effect between tasks was found.

The mean ±  standard deviation of the percentage of the relative phase of the in-phase (0° to ±  90°) and anti-phase (‒180° to ‒90° and 90° to 180°) were measured and compared by using a paired t-test to compare the relative phase between the percentages of the in-phase and anti-phase in both the slow- and fast-rhythm tasks. Statistical significance was set at p < 0.05 for all tests. Statistical analysis software (SPSS Statistics ver27, IBM Inc., Armonk, NY, USA) was used for all statistical analyses.

## Results

### Spatiotemporal variables of sprint running

A series of two-way repeated-measures analysis of variance revealed a significant interaction between tasks and intervals for sprint velocity (F (4.31, 90.41) =  15.89, p < 0.001), step rate (F (6.69, 140.58) =  8.04, p < 0.001), step length (F (6.59, 139.37) =  5.27, p < 0.001), stance time (F (5.73, 120.39) =  8.18, p < 0.001), and flight time (F (7.30, 153.32) =  4.23, p < 0.001) (Table 1) (S1 Table).

**Table 1:** Spatiotemporal variables of sprint running (n = 22).

		Intervals								Interaction (Tasks x Intervals)	
	**Tasks**	Start–5 m	5 m–10 m	10 m–15 m	15 m–20 m	20 m–25 m	25 m–30 m	30 m–35 m	35 m–40 m	F	P
**Sprint velocity (m/s)**	**Baseline**	4.17 ± 0.37	5.97 ± 0.42	6.63 ± 0.54	7.03 ± 0.70	7.22 ± 0.83	7.32 ± 0.88	7.38 ± 0.94	7.17 ± 1.04	15.89	< 0.001
	**Slow-rhythm**	3.77 ± 0.30	5.62 ± 0.44	6.10 ± 0.60	6.31 ± 0.75	6.35 ± 0.82	6.37 ± 0.88	6.37 ± 0.87	6.25 ± 0.91		
	**Fast-rhythm**	3.98 ± 0.24	5.98 ± 0.39	6.60 ± 0.55	7.00 ± 0.68	7.11 ± 0.72	7.17 ± 0.75	7.18 ± 0.74	7.10 ± 0.81		
**Step rate (Hz)**	**Baseline**	3.78 ± 0.27	4.07 ± 0.31	4.17 ± 0.35	4.19 ± 0.36	4.17 ± 0.37	4.13 ± 0.34	4.10 ± 0.35	3.86 ± 0.40	8.04	< 0.001
	**Slow-rhythm**	3.53 ± 0.32	3.67 ± 0.35	3.57 ± 0.35	3.53 ± 0.40	3.50 ± 0.39	3.46 ± 0.38	3.44 ± 0.33	3.48 ± 0.35		
	**Fast-rhythm**	3.96 ± 0.27	4.21 ± 0.31	4.25 ± 0.30	4.20 ± 0.25	4.14 ± 0.30	4.10 ± 0.25	4.06 ± 0.27	4.05 ± 0.31		
**Step length (m)**	**Baseline**	1.11 ± 0.14	1.47 ± 0.11	1.59 ± 0.11	1.68 ± 0.13	1.73 ± 0.15	1.77 ± 0.15	1.80 ± 0.18	1.86 ± 0.22	5.27	< 0.001
	**Slow-rhythm**	1.07 ± 0.08	1.54 ± 0.14	1.71 ± 0.16	1.79 ± 0.17	1.82 ± 0.20	1.84 ± 0.20	1.85 ± 0.20	1.80 ± 0.21		
	**Fast-rhythm**	1.01 ± 0.08	1.42 ± 0.10	1.55 ± 0.13	1.67 ± 0.13	1.72 ± 0.14	1.75 ± 0.14	1.77 ± 0.16	1.75 ± 0.17		
**Stance time (s)**	**Baseline**	0.184 ± 0.022	0.150 ± 0.021	0.140 ± 0.020	0.136 ± 0.020	0.135 ± 0.022	0.133 ± 0.023	0.137 ± 0.024	0.143 ± 0.027	8.18	< 0.001
	**Slow-rhythm**	0.187 ± 0.023	0.160 ± 0.024	0.156 ± 0.025	0.155 ± 0.026	0.155 ± 0.026	0.157 ± 0.028	0.158 ± 0.026	0.161 ± 0.027		
	**Fast-rhythm**	0.174 ± 0.021	0.144 ± 0.019	0.139 ± 0.020	0.138 ± 0.019	0.139 ± 0.021	0.139 ± 0.019	0.139 ± 0.018	0.142 ± 0.021		
**Flight time (s)**	**Baseline**	0.081 ± 0.020	0.097 ± 0.016	0.100 ± 0.015	0.104 ± 0.016	0.107 ± 0.016	0.111 ± 0.016	0.110 ± 0.016	0.120 ± 0.023	4.23	< 0.001
	**Slow-rhythm**	0.092 ± 0.017	0.114 ± 0.021	0.127 ± 0.019	0.132 ± 0.021	0.134 ± 0.022	0.136 ± 0.024	0.135 ± 0.023	0.131 ± 0.024		
	**Fast-rhythm**	0.074 ± 0.017	0.093 ± 0.019	0.097 ± 0.017	0.101 ± 0.015	0.104 ± 0.017	0.106 ± 0.016	0.108 ± 0.017	0.107 ± 0.017		

Multiple comparisons showed that sprint velocity in the slow-rhythm task significantly decreased from the baseline at all intervals (start – 5 m: 3.77 ±  0.30 m/s vs. 4.17 ±  0.37 m/s, p <  0.001, 5 m – 10 m: 5.62 ±  0.44 m/s vs. 5.97 ±  0.42 m/s, p <  0.001, 10 m – 15 m: 6.10 ±  0.60 m/s vs. 6.63 ±  0.54 m/s, p <  0.001, 15 m – 20 m: 6.31 ±  0.75 m/s vs. 7.03 ±  0.70 m/s, p <  0.001, 20 m –25 m: 6.35 ±  0.82 m/s vs. 7.22 ±  0.83 m/s, p <  0.001, 25 m – 30 m: 6.37 ±  0.88 m/s vs. 7.32 ±  0.88 m/s, p <  0.001, 30 m – 35 m: 6.37 ±  0.87 m/s vs. 7.38 ±  0.94 m/s, p <  0.001, 35 m – 40 m: 6.25 ±  0.91 m/s vs. 7.17 ±  1.04 m/s, p <  0.001). In the fast-rhythm task, sprint velocity decreased significantly from the baseline only in the initial 5 m interval (3.98 ±  0.24 m/s vs. 4.17 ±  0.37 m/s, p =  0.024) (S1 Table).

**Fig 2:**
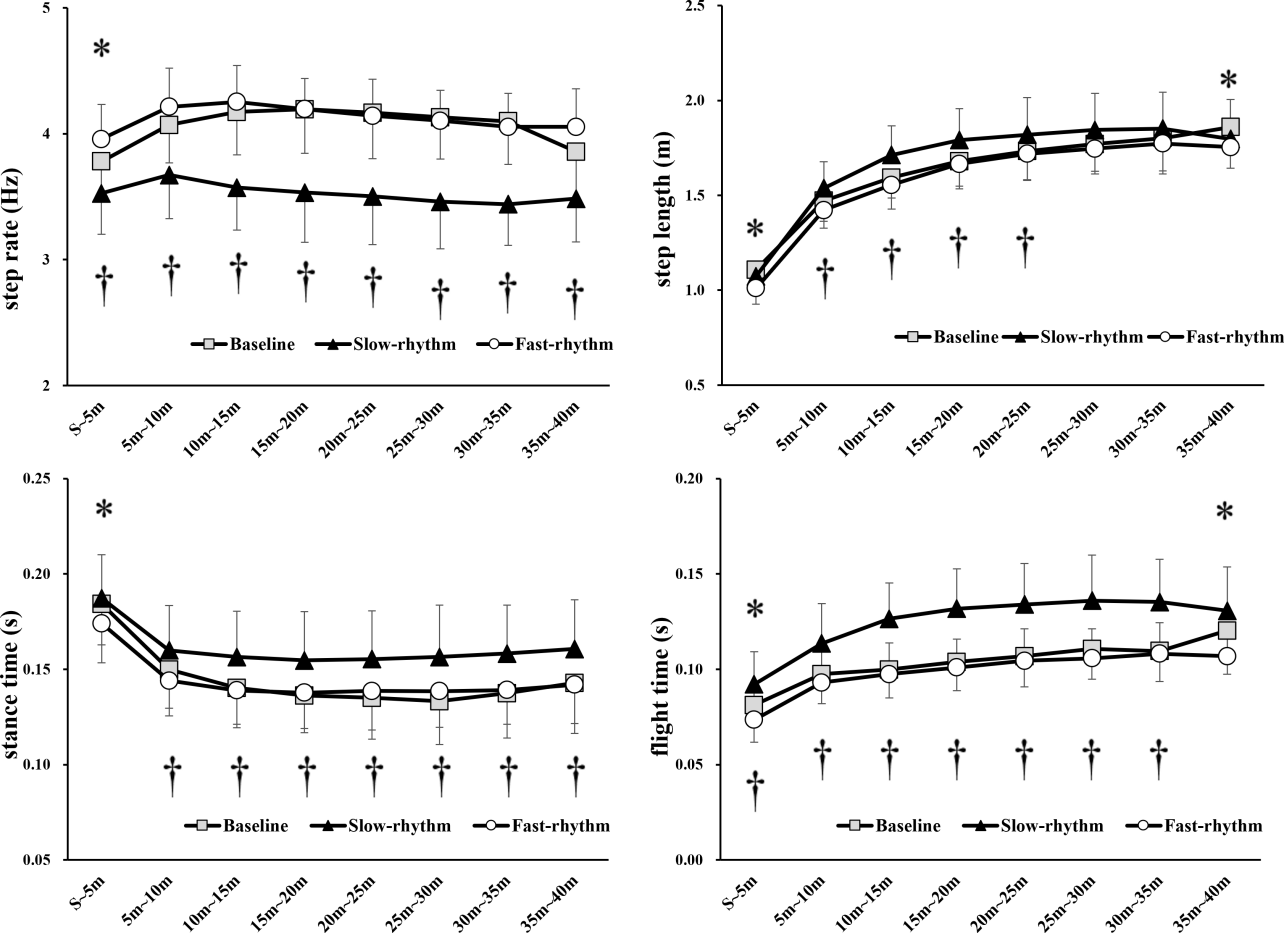
Comparison of step rate, step length, stance time, and flight time between tasks in eight intervals from the start to the 40 m mark. Two-way repeated-measures analysis of variance revealed a significant interaction between tasks (three tasks) and intervals (eight intervals). Baseline vs. fast-rhythm, * p <  0.05; baseline vs. slow-rhythm, †p <  0.05.

The step rate in the slow-rhythm task was significantly decreased compared to that during the baseline (3.53 ±  0.35 Hz vs. 3.78 ±  0.27 Hz, p <  0.001), while in the fast-rhythm task, it was significantly increased (3.96 ±  0.27 Hz vs. 3.78 ±  0.27 Hz, p =  0.001) from the start to the 5 m mark compared with that during the baseline. Furthermore, the step rate in the slow-rhythm task during the seven intervals of 5 m to 40 m was significantly lower than that at the baseline (5 – 10 m: 3.67 ±  0.35 Hz vs. 4.07 ±  0.31 Hz, p <  0.001; 10 – 15 m: 3.57 ±  0.35 Hz vs. 4.17 ±  0.35 Hz, p <  0.001; 15 – 20 m: 3.53 ±  0.40 Hz vs. 4.19 ±  0.36 Hz, p <  0.001; 20 – 25 m: 3.50 ±  0.39 Hz vs. 4.17 ±  0.37 Hz, p <  0.001; 25 – 30 m: 3.46 ±  0.38 Hz vs. 4.13 ±  0.34 Hz, p <  0.001; 30 – 35 m: 3.44 ±  0.33 Hz vs. 4.10 ±  0.35 Hz, p <  0.001; 35 – 40 m: 3.48 ±  0.35 Hz vs. 3.86 ±  0.40 Hz, p <  0.001) (Fig 2).

The step length in the slow-rhythm task increased significantly in the 5 – 25 m interval (5 – 10 m: 1.54 ±  0.14 m vs. 1.47 ±  0.11 m, p =  0.043; 10 – 15 m: 1.71 ±  0.16 m vs. 1.59 ±  0.11 m, p <  0.001; 15 – 20 m: 1.79 ±  0.17 m vs. 1.68 ±  0.13 m, p =  0.001; 20 – 25 m: 1.82 ±  0.20 m vs. 1.73 ±  0.15 m, p = 0.032;), while in the fast-rhythm task it decreased from the start – 5 m interval (1.01 ±  0.85 m vs. 1.11 ±  0.14 m, p <  0.001) and from the 35 – 40 m mark compared to baseline (1.75 ±  0.17 m vs. 1.86 ±  0.22 m, p =  0.046) (Fig 2).

In seven intervals from the 5 m to 40 m mark, the stance time in the slow-rhythm task was significantly higher than during the baseline (5 – 10 m: 0.160 ±  0.024 s vs. 0.150 ±  0.021 s, p =  0.020; 10 – 15 m: 0.156 ±  0.025 s vs. 0.140 ±  0.020 s, p <  0.001; 15 – 20 m: 0.155 ±  0.026 s vs. 0.136 ±  0.020 s, p <  0.001; 20 – 25 m: 0.155 ±  0.026 s vs. 0.135 ±  0.022 s, p <  0.001; 25 – 30 m: 0.157 ±  0.028 s vs. 0.133 ±  0.023 s, p <  0.001; 30 – 35 m: 0.158 ±  0.026 s vs. 0.137 ±  0.024 s, p <  0.001; 35 – 40 m: 0.161 ±  0.027 s vs. 0.143 ±  0.027 s, p <  0.001). Furthermore, in the slow-rhythm task, the stance time significantly increased when the step rate decreased compared to the baseline. The stance time in the fast-rhythm task decreased significantly from the start to the 5 m mark (0.174 ±  0.021 s vs. 0.184 ±  0.022 s, p =  0.017) compared to that at baseline (Fig 2).

The flight time in the fast-rhythm task decreased significantly, while the flight time in the slow-rhythm task significantly increased from the start to the 5 m mark (fast-rhythm: 0.074 ±  0.017 s vs. 0.081 ±  0.020 s, p =  0.046; slow-rhythm: 0.092 ±  0.017 s vs. 0.081 ±  0.020 s, p =  0.007) compared to that baseline. The flight time of the slow-rhythm task in the six intervals from the 5 m to 35 m mark significantly increased compared to the corresponding baseline values (5 – 10 m: 0.114 ±  0.021 s vs. 0.097 ±  0.016 s, p <  0.001; 10 – 15 m: 0.127 ±  0.019 s vs. 0.100 ±  0.015 s, p <  0.001; 15 – 20 m: 0.132 ±  0.021 s vs. 0.104 ±  0.016 s, p <  0.001; 20 – 25 m: 0.134 ±  0.022 s vs. 0.107 ±  0.016 s, p <  0.001; 25 – 30 m: 0.136 ±  0.024 s vs. 0.111 ±  0.016 s, p <  0.001; 30 – 35 m: 0.135 ±  0.023 s vs. 0.110 ±  0.016 s, p <  0.001). The flight time of the fast-rhythm task from the 35 m to 40 m mark was significantly decreased compared to that at baseline (0.107 ±  0.017 s vs. 0.120 ±  0.023 s, p =  0.033) (Fig 2).

### The relative phase of the in-phase and anti-phase

**Fig 3:**
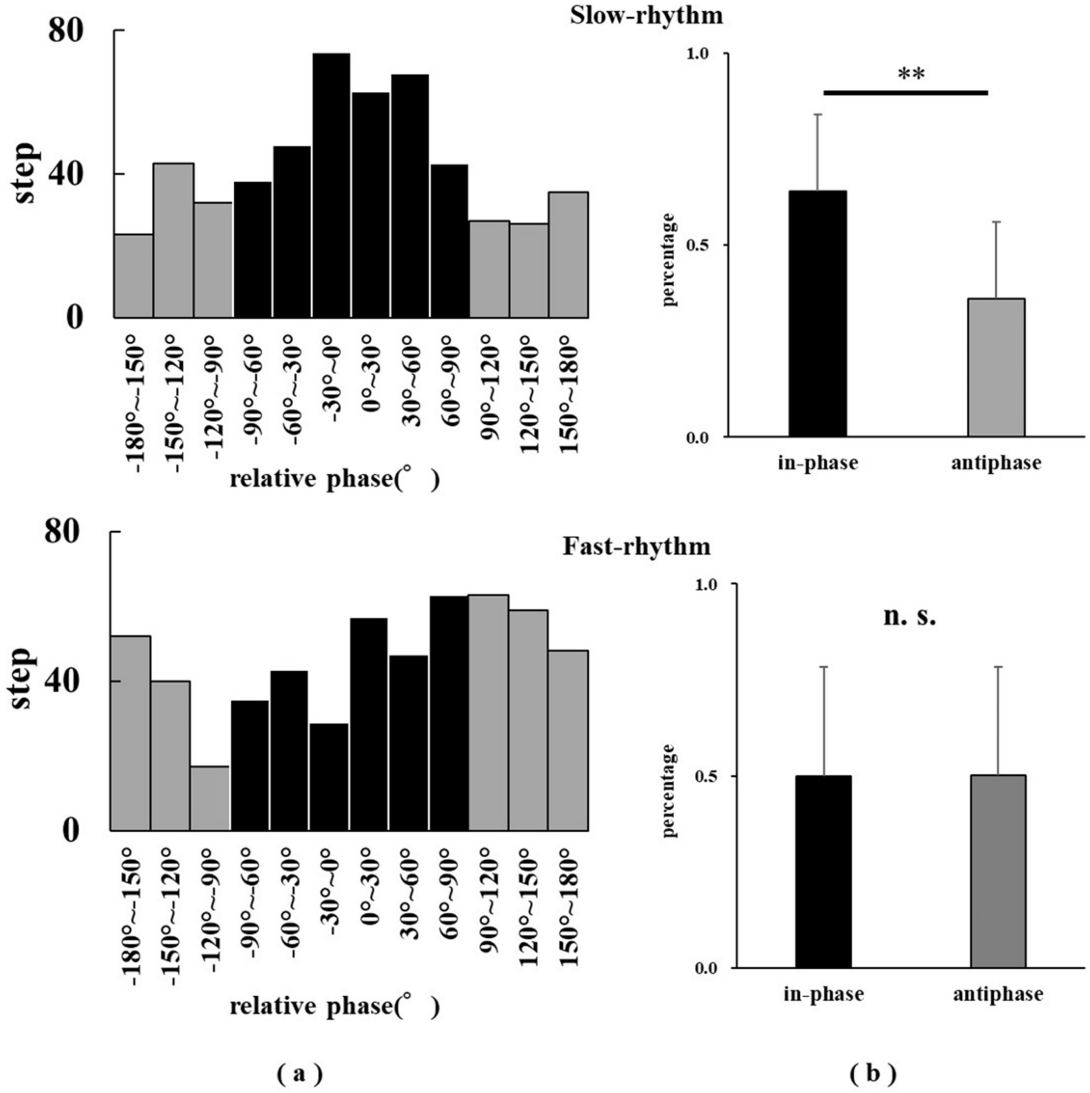
(a) Histogram of relative phase at all steps for all participants. (b) Comparison of the relative phase between the percentages of in-phase and anti-phase in both slow- and fast-rhythm tasks. A paired t-test revealed a significant difference between in-phase and anti-phase in the slow-rhythm task (**p <  0.01). No significant differences were observed between the percentages of the in-phase and anti-phase during the fast-rhythm task (n. s., not significant).

The paired-sample t-test revealed that the relative phase was higher in the percentages of the in-phase than the anti-phase in the slow-rhythm task (t (21) =  3.18, p =  0.005). However, no significant differences were observed between the percentages of in-phase and anti-phase during the fast-rhythm task (t (21) =  ‒0.01, p =  0.99) (Fig 3) (S2 Table).

## Discussion

We aimed to investigate the effects of auditory guidance on the spatiotemporal variables of high-velocity sprint running in junior high school students. We hypothesized that the step rate in high-velocity sprint running can be modified by synchronizing the motion with a rhythmic auditory guide. Our findings showed that slow-rhythm auditory guidance decreases step rate while increasing step length, whereas fast-rhythm guidance produces the opposite effect: increasing step rate and reducing step length. These results suggest that rhythmic auditory guides could serve as valuable tools for coaching and optimizing sprint performance by targeting specific spatiotemporal variables.

Rhythmic manipulation of sprint running by metronome

The step rate in the slow-rhythm task significantly decreased compared to the corresponding baseline in all intervals from the start to the 40 m mark. The step rate in the fast-rhythm task significantly increased from the start to the 5 m mark compared to the baseline. Several studies [6,9,12,25–27] have indicated that the tempo of movement can be manipulated using an auditory guide. These results suggest that a metronome may effectively manipulate the tempo of submaximal sprint running. Furthermore, step length increased significantly with a change in step rate in the slow-rhythm task. Hence, sprint running was transformed into step rate in the fast-rhythm task and step length in the slow-rhythm task. These results support our hypothesis that the step rate in high-velocity sprint running can be modified by synchronizing the motion with a rhythmic auditory guide.

Conventional training to improve step rate in sprint running often involves marked running [28]. However, this type of training, which focuses on marks, tends to limit step length because of the fixed distance between marks [28]. This limitation makes it difficult to enhance both step rate and step length simultaneously. Since step rate and step length negatively interact [29], adopting training strategies that can improve or maintain both aspects concurrently to boost running velocity is essential. Utilizing auditory guides in training may provide the advantage of not restricting step length, thus allowing for simultaneous improvements in step length and step rate [12]. The results of this study will be useful for future coaching settings.

### Effect of running skills

#### The slow-rhythm task.

The step rate in the slow-rhythm task decreased significantly compared to that at the baseline. The step rate is calculated based on the number of steps per second and consists of the stance and flight times [29]. Therefore, to decrease the step rate, increasing either or both the stance and flight times is necessary. In this study, increases in stance and flight times were observed during intervals where the step rate significantly decreased. Thus, the decrease in step rate during the slow-rhythm task observed in this study may be because of increased stance and flight times.

The step length in the slow-rhythm task from the 5 m mark to the 25 m mark significantly increased compared to that during the baseline. Step length is composed of two components—stance distance and flight distance—and a strong positive correlation between flight distance and step length has been found [29]. An increase in flight distance can be obtained by increasing the horizontal and vertical impulses and the stance time and flight time [19,29–31]. Impulse is obtained by integrating the force with time. Even if the force exerted against the ground is small, increasing the ground stance time increases the force product, increasing step length. Therefore, it is believed that the participants (age: 13.18 ±  0.65 years) in this study could increase their force product and obtain a higher step length by increasing the stance time they were on the ground.

A 100 m run consists of an acceleration phase, constant velocity phase, and deceleration phase [32]. The acceleration phase is important for reaching the velocity [33]. Previous studies [34, 35] have shown that increasing the step length contributes to adjusting the sprint velocity in lower velocity ranges, whereas increasing the step rate contributes to increasing the sprint velocity in higher velocity ranges. Therefore, it is necessary to increase the step length during the acceleration phase to generate acceleration. Considering that the slow-rhythm task increased the step length in the acceleration phase (up to the 25 m mark), slow-rhythm auditory guide training may help increase step length in the acceleration phase.

#### The fast-rhythm task.

From the start to the 5 m mark, the step rate significantly increased in the fast-rhythm task compared to the baseline. This increase in step rate indicates that, during the fast-rhythm task, the tempo of sprint running was effectively manipulated through an auditory guide, as seen in the slow-rhythm task. Compared with the baseline, flight and stance times significantly decreased in the interval where the step rate increased significantly (Fig 2: S – 5 m). This result is consistent with the findings of several studies that reported that decreases in stance and flight time can be attributed to increases in the step rate [19,29,30]. The increase in step rate observed in this study may be because of decreased stance and flight times.

In the fast-rhythm task, compared to the baseline, there was a significant increase in step rate in the S-5m section, where the step length significantly decreased. The step rate and step length, which determine the running velocity, negatively interact [29]. Therefore, an increase in one results in a decrease in the other. The increase in step rate and decrease in step length observed in this study indicate that these negative interactions occurred. This result implies a shift from a default sprint running motion to a step rate-dominant sprint running motion by synchronizing with a fast-rhythm.

### Synchronization of sprint running and the metronome

#### The relative phase of the in-phase and anti-phase.

The participants had no instructions on when to synchronize with the auditory guide (e.g., synchronize the sound with ground contact, synchronize the sound with arm swing, etc.). Therefore, the participants ran with the auditory guide based on their rhythm. However, the in-phase ratio was significantly higher than the anti-phase ratio during the slow-rhythm task. This difference indicates that the contact timing during sprint running was synchronized well with the metronome auditory guide.

In several studies, simple cyclic movements, such as finger tapping [26,36] and knee flexion and extension [37], in response to external stimuli with a rhythm exhibited greater stability during in-phase coordination than during the anti-phase. Furthermore, the unintentional phase entrainment phenomenon from the anti-phase to the in-phase has been widely reported [26,36,37]. Thus, in-phase entrainment occurs even during sprint running, similar to simple motor tasks [26,36,37].

When using a metronome during a slow rhythm for sprint running, instructions to synchronize the moment of touchdown and the metronome auditory guide may make the change in the running rhythm more stable. However, no significant differences were observed between the in-phase and anti-phase in the fast-rhythm task (Fig 3). Deviations from the intended phase relation and enhanced fluctuations have been observed at higher beat rates [38]. Furthermore, sprint velocity was significantly lower than that during the baseline for the slow-rhythm task (starting at the 40 m mark) and did not differ from that during the baseline for the fast-rhythm task (5–40 m). Thus, the participants may have synchronized to the intended rhythmic auditory guide by decreasing sprint velocity in the slow-rhythm task. However, in the fast-rhythm task, no significant differences were observed between the in-phase and anti-phase (Fig 3), and the participants might have performed sprint running at a high velocity, indicating that they may not have been able to synchronize the auditory guide with the intended running rhythm.

#### The slow-rhythm task.

In this study, we exposed participants to auditory stimulation with a slow rhythm (‒10%) compared to their natural running rhythm (baseline) and tasked them to synchronize. Therefore, there was a significant decrease in the step rate of sprint running throughout the entire distance from start to finish. This decrease in step rate indicates that synchronization with a slow rhythm is possible even during sprint running (Fig 2). Numerous studies have reported a significant reduction in the rhythm of movement when listening to slow-rhythm music [15, 16].

#### The fast-rhythm task.

The sprint velocity significantly decreased from baseline to the 5 m mark in the fast-rhythm task. Participants were instructed to synchronize with the metronome, which led them to reduce their sprint velocity immediately after starting. A previous study investigated responses to auditory stimuli rhythms during low-velocity running exercises [12], comparing “instructed” synchronization with music rhythm and “spontaneous entrainment,” where synchronization occurred unconsciously. The results showed that, under spontaneous entrainment, participants improved in all metrics: step rate, step length, and sprint velocity. In contrast, only an increase in step rate was observed under the instructed condition. This result is explained by Rejeski’s parallel processing model [13], in which physiological cues (e.g., heart rate, respiration rate) become dominant as the intensity of exercise increases [12]. The perception of neural signs of effort from muscles, joints, and the cardiopulmonary system increases as exercise becomes more demanding, drawing attention to the exercise’s painful and fatiguing effects [12, 13]. Therefore, our study’s observed decrease in sprint velocity may be attributed to the participants consciously synchronizing the music tempo with their running tempo, leading to divided attention. Accordingly, sprint velocity may improve if participants do not consciously focus on auditory stimuli, thus allowing for unconscious synchronization. Further studies are required to explore this possibility.

## Limitations

During this trial, we instructed the participants to synchronize their running with the metronome, wherein the auditory guide was played from start to finish. This study’s constant-frequency metronome auditory guide modified the step rate from the start to the 5 m mark during the baseline trial. However, step rate and step length indicate temporal changes that increase with sprint velocity [35,39,40]. There is also a threshold of synchronous tempo in music, called the entrainment basin [15]. It is expected that there are tempo frequencies that can be synchronized between sprint running and music tempos. Therefore, future studies should explore experiments where the music tempo is adjusted to align with the acceleration and maximum velocity phases.

In this study, we recruited male and female junior high school students who belonged to a track and field club. Reportedly, females are more likely to have their exercise tempo influenced by music tempo than males [15]. Therefore, there may be sex-based differences in the influence of music. Furthermore responses to music during running differ depending on the individual’s exercise experience. Unskilled participants are more affected by music than skilled participants since skilled participants tend to concentrate more on the exercise task [15,41]. Therefore, future research is needed to determine the applicability of this study’s findings to sprint running in other athlete populations, such as elite and college athletes, and to examine the differences between male and female participants.

## Conclusions

This study examined the effects of auditory stimuli using a metronome on sprint running in junior high school students. We found that the auditory guide could manipulate these students’ step rate and step length. It is expected that training using auditory stimuli will be used in coaching in sprint running.

## Supporting Information

S1 Table Spatiotemporal variables of sprint running for each participant. (DOCX)

S2 Table Relative phase between in-phase and anti-phase percentages in slow and fast rhythm tasks for each participant. (DOCX)
