# Supplementary material for: Effects of rhythmic auditory guide on sprint running
Source: PLoS One. 2025 Mar 21;20(3):e0319738. doi: 10.1371/journal.pone.0319738 (PMC11927899; doi:10.1371/journal.pone.0319738)
Supplement: S1 Table — (DOCX) [file pone.0319738.s001.docx]

## **Supporting information**

**S1 Table. Spatiotemporal variables of sprint running for each participant**

| **Baseline** | **S–5m** |  |  |  |  | **5m–10m** |  |  |  |  | **10m–15m** |  |  |  |  | **15m–20m** |  |  |  |  | **20m - 25m** |  |  |  |  | **25m - 30m** |  |  |  |  | **30m - 35m** |  |  |  |  | **35m - 40m** |  |  |  |  |
| --- | --- | --- | --- | --- | --- | --- | --- | --- | --- | --- | --- | --- | --- | --- | --- | --- | --- | --- | --- | --- | --- | --- | --- | --- | --- | --- | --- | --- | --- | --- | --- | --- | --- | --- | --- | --- | --- | --- | --- | --- |
|  | **Sprint velocity** | **Step length** | **Step rate** | **Stance time** | **Flight time** | **Sprint velocity** | **Step length** | **Step rate** | **Stance time** | **Flight time** | **Sprint velocity** | **Step length** | **Step rate** | **Stance time** | **Flight time** | **Sprint velocity** | **Step length** | **Step rate** | **Stance time** | **Flight time** | **Sprint velocity** | **Step length** | **Step rate** | **Stance time** | **Flight time** | **Sprint velocity** | **Step length** | **Step rate** | **Stance time** | **Flight time** | **Sprint velocity** | **Step length** | **Step rate** | **Stance time** | **Flight time** | **Sprint velocity** | **Step length** | **Step rate** | **Stance time** | **Flight time** |
|  | **4.26** | **1.09** | **3.91** | **0.181** | **0.068** | **6.32** | **1.51** | **4.17** | **0.138** | **0.100** | **7.06** | **1.65** | **4.29** | **0.136** | **0.096** | **7.36** | **1.73** | **4.25** | **0.128** | **0.108** | **7.64** | **1.80** | **4.25** | **0.124** | **0.113** | **7.79** | **1.87** | **4.17** | **0.121** | **0.117** | **8.05** | **1.91** | **4.21** | **0.119** | **0.121** | **7.84** | **1.91** | **4.10** | **0.126** | **0.119** |
|  | **3.96** | **0.97** | **4.09** | **0.174** | **0.085** | **6.00** | **1.39** | **4.32** | **0.178** | **0.098** | **6.78** | **1.55** | **4.36** | **0.125** | **0.104** | **7.06** | **1.62** | **4.36** | **0.129** | **0.102** | **7.02** | **1.64** | **4.29** | **0.128** | **0.104** | **7.19** | **1.69** | **4.25** | **0.126** | **0.106** | **7.23** | **1.70** | **4.25** | **0.132** | **0.106** | **6.98** | **1.74** | **4.00** | **0.142** | **0.115** |
|  | **4.35** | **1.14** | **3.83** | **0.160** | **0.094** | **5.85** | **1.33** | **4.40** | **0.138** | **0.090** | **6.67** | **1.50** | **4.44** | **0.131** | **0.092** | **7.27** | **1.70** | **4.29** | **0.124** | **0.108** | **7.59** | **1.72** | **4.40** | **0.121** | **0.106** | **7.64** | **1.77** | **4.32** | **0.119** | **0.110** | **7.69** | **1.78** | **4.32** | **0.125** | **0.108** | **7.69** | **1.81** | **4.25** | **0.121** | **0.113** |
|  | **4.46** | **1.16** | **3.83** | **0.150** | **0.104** | **5.94** | **1.41** | **4.21** | **0.128** | **0.110** | **6.78** | **1.54** | **4.40** | **0.131** | **0.096** | **7.06** | **1.59** | **4.44** | **0.124** | **0.104** | **7.32** | **1.66** | **4.40** | **0.117** | **0.110** | **7.23** | **1.67** | **4.32** | **0.122** | **0.110** | **7.36** | **1.72** | **4.29** | **0.117** | **0.117** | **7.27** | **1.80** | **4.03** | **0.126** | **0.123** |
|  | **3.75** | **0.94** | **3.98** | **0.186** | **0.069** | **5.45** | **1.31** | **4.17** | **0.143** | **0.092** | **6.28** | **1.44** | **4.36** | **0.136** | **0.092** | **6.45** | **1.52** | **4.25** | **0.129** | **0.104** | **6.52** | **1.55** | **4.21** | **0.131** | **0.108** | **6.78** | **1.58** | **4.29** | **0.128** | **0.106** | **6.78** | **1.64** | **4.14** | **0.131** | **0.113** | **6.78** | **1.69** | **4.00** | **0.133** | **0.119** |
|  | **3.74** | **1.01** | **3.69** | **0.208** | **0.061** | **5.48** | **1.45** | **3.78** | **0.151** | **0.110** | **5.91** | **1.60** | **3.69** | **0.151** | **0.119** | **6.15** | **1.64** | **3.75** | **0.149** | **0.117** | **6.28** | **1.69** | **3.72** | **0.147** | **0.119** | **6.32** | **1.71** | **3.69** | **0.151** | **0.119** | **6.38** | **1.74** | **3.66** | **0.151** | **0.121** | **6.15** | **1.73** | **3.56** | **0.158** | **0.125** |
|  | **3.31** | **0.93** | **3.56** | **0.239** | **0.047** | **5.06** | **1.32** | **3.84** | **0.201** | **0.060** | **5.13** | **1.39** | **3.69** | **0.192** | **0.075** | **5.08** | **1.35** | **3.78** | **0.194** | **0.073** | **4.90** | **1.36** | **3.61** | **0.204** | **0.073** | **4.86** | **1.37** | **3.56** | **0.203** | **0.081** | **4.69** | **1.30** | **3.61** | **0.210** | **0.071** | **4.24** | **1.24** | **3.43** | **0.228** | **0.067** |
|  | **4.20** | **1.24** | **3.40** | **0.190** | **0.099** | **5.71** | **1.62** | **3.53** | **0.163** | **0.121** | **6.38** | **1.81** | **3.53** | **0.150** | **0.133** | **6.59** | **1.84** | **3.58** | **0.139** | **0.140** | **6.74** | **1.92** | **3.50** | **0.142** | **0.142** | **6.78** | **1.84** | **3.69** | **0.140** | **0.131** | **7.02** | **1.90** | **3.69** | **0.138** | **0.133** | **6.78** | **1.89** | **3.58** | **0.136** | **0.142** |
|  | **3.91** | **1.01** | **3.87** | **0.218** | **0.056** | **5.71** | **1.43** | **4.00** | **0.165** | **0.081** | **6.25** | **1.51** | **4.14** | **0.158** | **0.085** | **6.42** | **1.63** | **3.93** | **0.158** | **0.094** | **6.38** | **1.56** | **4.10** | **0.160** | **0.085** | **6.49** | **1.76** | **3.69** | **0.157** | **0.110** | **6.42** | **1.78** | **3.61** | **0.168** | **0.117** | **5.80** | **1.85** | **3.14** | **0.175** | **0.144** |
|  | **3.66** | **1.05** | **3.48** | **0.209** | **0.086** | **5.29** | **1.44** | **3.66** | **0.172** | **0.100** | **5.80** | **1.58** | **3.66** | **0.172** | **0.096** | **6.00** | **1.64** | **3.66** | **0.167** | **0.104** | **6.28** | **1.64** | **3.84** | **0.164** | **0.098** | **6.32** | **1.68** | **3.75** | **0.164** | **0.102** | **6.28** | **1.66** | **3.78** | **0.164** | **0.102** | **5.85** | **1.63** | **3.58** | **0.178** | **0.108** |
|  | **4.24** | **1.11** | **3.81** | **0.200** | **0.051** | **6.15** | **1.44** | **4.29** | **0.160** | **0.073** | **6.56** | **1.57** | **4.17** | **0.150** | **0.085** | **7.02** | **1.59** | **4.40** | **0.140** | **0.090** | **7.02** | **1.68** | **4.17** | **0.136** | **0.104** | **7.06** | **1.71** | **4.14** | **0.135** | **0.104** | **7.14** | **1.71** | **4.17** | **0.143** | **0.098** | **6.90** | **1.71** | **4.03** | **0.143** | **0.106** |
|  | **4.35** | **1.06** | **4.09** | **0.174** | **0.079** | **6.82** | **1.56** | **4.36** | **0.133** | **0.096** | **7.32** | **1.74** | **4.21** | **0.119** | **0.117** | **7.95** | **1.87** | **4.25** | **0.114** | **0.121** | **8.39** | **1.98** | **4.25** | **0.110** | **0.127** | **8.51** | **1.99** | **4.29** | **0.110** | **0.121** | **8.76** | **1.99** | **4.40** | **0.108** | **0.119** | **8.63** | **2.05** | **4.21** | **0.110** | **0.127** |
|  | **4.15** | **1.16** | **3.56** | **0.180** | **0.099** | **5.91** | **1.59** | **3.72** | **0.150** | **0.119** | **6.70** | **1.80** | **3.72** | **0.142** | **0.125** | **7.27** | **1.86** | **3.90** | **0.138** | **0.119** | **7.41** | **1.91** | **3.87** | **0.138** | **0.121** | **7.59** | **1.98** | **3.84** | **0.114** | **0.160** | **7.79** | **1.96** | **3.97** | **0.142** | **0.113** | **7.36** | **2.09** | **3.53** | **0.150** | **0.135** |
|  | **3.79** | **1.01** | **3.75** | **0.170** | **0.118** | **6.28** | **1.45** | **4.32** | **0.119** | **0.108** | **7.50** | **1.66** | **4.53** | **0.113** | **0.108** | **8.11** | **1.76** | **4.62** | **0.113** | **0.104** | **8.33** | **1.86** | **4.49** | **0.111** | **0.113** | **8.63** | **1.92** | **4.49** | **0.113** | **0.110** | **8.51** | **1.95** | **4.36** | **0.115** | **0.115** | **8.05** | **2.32** | **3.48** | **0.124** | **0.165** |
|  | **4.43** | **1.08** | **4.11** | **0.179** | **0.072** | **6.49** | **1.50** | **4.32** | **0.142** | **0.088** | **7.19** | **1.56** | **4.62** | **0.133** | **0.085** | **7.74** | **1.71** | **4.53** | **0.133** | **0.088** | **8.00** | **1.70** | **4.71** | **0.132** | **0.085** | **8.16** | **1.82** | **4.49** | **0.126** | **0.098** | **8.28** | **1.93** | **4.29** | **0.140** | **0.092** | **8.05** | **1.91** | **4.21** | **0.143** | **0.096** |
|  | **4.23** | **1.00** | **4.24** | **0.154** | **0.078** | **6.25** | **1.43** | **4.36** | **0.136** | **0.092** | **6.67** | **1.56** | **4.29** | **0.128** | **0.104** | **6.98** | **1.64** | **4.25** | **0.125** | **0.110** | **7.02** | **1.70** | **4.14** | **0.129** | **0.113** | **7.06** | **1.75** | **4.03** | **0.133** | **0.110** | **7.06** | **1.72** | **4.10** | **0.129** | **0.117** | **6.78** | **1.96** | **3.45** | **0.139** | **0.154** |
|  | **4.58** | **1.27** | **3.60** | **0.186** | **0.068** | **6.03** | **1.78** | **3.38** | **0.171** | **0.119** | **6.59** | **1.70** | **3.87** | **0.158** | **0.100** | **7.02** | **1.84** | **3.81** | **0.154** | **0.108** | **7.02** | **1.96** | **3.58** | **0.158** | **0.121** | **7.14** | **1.82** | **3.93** | **0.156** | **0.098** | **7.19** | **1.92** | **3.75** | **0.161** | **0.108** | **6.90** | **2.00** | **3.45** | **0.164** | **0.123** |
|  | **4.71** | **1.35** | **3.50** | **0.182** | **0.099** | **6.19** | **1.61** | **3.84** | **0.161** | **0.098** | **6.78** | **1.75** | **3.87** | **0.146** | **0.110** | **7.27** | **1.92** | **3.78** | **0.142** | **0.123** | **7.50** | **1.95** | **3.84** | **0.138** | **0.123** | **7.64** | **2.04** | **3.75** | **0.139** | **0.127** | **7.59** | **2.15** | **3.53** | **0.142** | **0.144** | **7.50** | **2.23** | **3.36** | **0.144** | **0.154** |
|  | **3.96** | **0.97** | **4.07** | **0.181** | **0.067** | **6.03** | **1.44** | **4.17** | **0.153** | **0.081** | **6.63** | **1.53** | **4.32** | **0.151** | **0.077** | **7.10** | **1.57** | **4.53** | **0.143** | **0.081** | **7.32** | **1.69** | **4.32** | **0.139** | **0.088** | **7.27** | **1.65** | **4.40** | **0.146** | **0.085** | **7.32** | **1.69** | **4.32** | **0.142** | **0.088** | **7.19** | **1.71** | **4.21** | **0.153** | **0.083** |
|  | **4.20** | **1.08** | **3.90** | **0.147** | **0.104** | **6.42** | **1.48** | **4.32** | **0.108** | **0.121** | **7.10** | **1.57** | **4.53** | **0.110** | **0.113** | **7.59** | **1.66** | **4.57** | **0.104** | **0.113** | **7.89** | **1.73** | **4.57** | **0.104** | **0.115** | **8.05** | **1.74** | **4.62** | **0.099** | **0.119** | **8.22** | **1.85** | **4.44** | **0.101** | **0.125** | **8.28** | **1.86** | **4.44** | **0.100** | **0.123** |
|  | **4.60** | **1.46** | **3.14** | **0.194** | **0.110** | **5.61** | **1.41** | **3.97** | **0.149** | **0.100** | **6.78** | **1.54** | **4.40** | **0.133** | **0.092** | **7.59** | **1.68** | **4.53** | **0.129** | **0.090** | **8.28** | **1.79** | **4.62** | **0.125** | **0.092** | **8.51** | **1.84** | **4.62** | **0.117** | **0.102** | **8.63** | **1.96** | **4.40** | **0.131** | **0.094** | **8.63** | **1.96** | **4.40** | **0.128** | **0.100** |
|  | **4.84** | **1.30** | **3.74** | **0.191** | **0.072** | **6.35** | **1.46** | **4.36** | **0.139** | **0.088** | **7.10** | **1.51** | **4.71** | **0.119** | **0.092** | **7.64** | **1.59** | **4.80** | **0.122** | **0.085** | **7.89** | **1.64** | **4.80** | **0.115** | **0.092** | **7.95** | **1.75** | **4.53** | **0.113** | **0.106** | **8.05** | **1.66** | **4.85** | **0.114** | **0.092** | **8.00** | **1.82** | **4.40** | **0.121** | **0.106** |
| **Slow-rhythm** | **S–5m** |  |  |  |  | **5m–10m** |  |  |  |  | **10m–15m** |  |  |  |  | **15m–20m** |  |  |  |  | **20m–25m** |  |  |  |  | **25m - 30m** |  |  |  |  | **30m - 35m** |  |  |  |  | **35m - 40m** |  |  |  |  |
|  | **Sprint velocity** | **Step length** | **Step rate** | **Stance time** | **Flight time** | **Sprint velocity** | **Step length** | **Step rate** | **Stance time** | **Flight time** | **Sprint velocity** | **Step length** | **Step rate** | **Stance time** | **Flight time** | **Sprint velocity** | **Step length** | **Step rate** | **Stance time** | **Flight time** | **Sprint velocity** | **Step length** | **Step rate** | **Stance time** | **Flight time** | **Sprint velocity** | **Step length** | **Step rate** | **Stance time** | **Flight time** | **Sprint velocity** | **Step length** | **Step rate** | **Stance time** | **Flight time** | **Sprint velocity** | **Step length** | **Step rate** | **Stance time** | **Flight time** |
|  | **4.14** | **1.10** | **3.77** | **0.172** | **0.093** | **6.19** | **1.62** | **3.81** | **0.144** | **0.113** | **6.70** | **1.73** | **3.87** | **0.133** | **0.125** | **7.19** | **1.81** | **3.97** | **0.126** | **0.125** | **7.36** | **1.99** | **3.69** | **0.129** | **0.142** | **7.27** | **2.05** | **3.56** | **0.133** | **0.148** | **7.32** | **2.03** | **3.61** | **0.135** | **0.140** | **7.32** | **1.81** | **4.03** | **0.131** | **0.119** |
|  | **3.88** | **0.95** | **4.09** | **0.175** | **0.068** | **5.56** | **1.35** | **4.10** | **0.150** | **0.094** | **6.12** | **1.62** | **3.78** | **0.151** | **0.115** | **6.28** | **1.66** | **3.78** | **0.153** | **0.113** | **6.45** | **1.79** | **3.61** | **0.150** | **0.125** | **6.32** | **1.71** | **3.69** | **0.147** | **0.125** | **6.45** | **1.87** | **3.45** | **0.151** | **0.138** | **6.22** | **1.63** | **3.81** | **0.149** | **0.115** |
|  | **4.26** | **1.19** | **3.58** | **0.166** | **0.106** | **5.33** | **1.50** | **3.56** | **0.144** | **0.135** | **5.97** | **1.68** | **3.56** | **0.139** | **0.142** | **6.28** | **1.78** | **3.53** | **0.138** | **0.146** | **6.35** | **1.85** | **3.43** | **0.139** | **0.152** | **6.35** | **1.83** | **3.48** | **0.143** | **0.148** | **6.35** | **1.88** | **3.38** | **0.143** | **0.154** | **6.28** | **1.86** | **3.38** | **0.142** | **0.154** |
|  | **3.85** | **1.13** | **3.41** | **0.172** | **0.114** | **5.80** | **1.52** | **3.81** | **0.139** | **0.125** | **6.49** | **1.76** | **3.69** | **0.140** | **0.133** | **6.63** | **1.95** | **3.40** | **0.133** | **0.163** | **6.63** | **2.11** | **3.14** | **0.139** | **0.177** | **6.59** | **1.95** | **3.38** | **0.132** | **0.158** | **6.78** | **1.95** | **3.48** | **0.135** | **0.158** | **6.52** | **1.98** | **3.29** | **0.140** | **0.167** |
|  | **3.76** | **1.08** | **3.50** | **0.185** | **0.097** | **5.41** | **1.50** | **3.61** | **0.156** | **0.119** | **5.77** | **1.60** | **3.61** | **0.153** | **0.125** | **6.03** | **1.71** | **3.53** | **0.146** | **0.135** | **6.25** | **1.80** | **3.48** | **0.147** | **0.142** | **6.28** | **1.81** | **3.48** | **0.142** | **0.146** | **6.45** | **1.77** | **3.64** | **0.142** | **0.133** | **6.32** | **1.78** | **3.56** | **0.147** | **0.138** |
|  | **3.76** | **1.09** | **3.46** | **0.191** | **0.086** | **5.22** | **1.41** | **3.69** | **0.161** | **0.113** | **5.80** | **1.59** | **3.64** | **0.160** | **0.117** | **5.80** | **1.79** | **3.24** | **0.161** | **0.146** | **5.83** | **1.74** | **3.36** | **0.161** | **0.135** | **5.91** | **1.87** | **3.16** | **0.163** | **0.156** | **5.71** | **1.80** | **3.18** | **0.164** | **0.152** | **5.48** | **1.71** | **3.20** | **0.175** | **0.138** |
|  | **3.24** | **0.96** | **3.36** | **0.225** | **0.063** | **4.49** | **1.26** | **3.56** | **0.208** | **0.077** | **4.62** | **1.40** | **3.29** | **0.207** | **0.098** | **4.51** | **1.42** | **3.18** | **0.206** | **0.110** | **4.27** | **1.36** | **3.14** | **0.211** | **0.108** | **4.27** | **1.34** | **3.18** | **0.229** | **0.085** | **4.33** | **1.29** | **3.36** | **0.221** | **0.079** | **4.14** | **1.26** | **3.29** | **0.229** | **0.077** |
|  | **3.88** | **1.13** | **3.44** | **0.195** | **0.090** | **5.69** | **1.88** | **3.02** | **0.164** | **0.163** | **5.88** | **1.80** | **3.27** | **0.158** | **0.152** | **5.97** | **2.11** | **2.82** | **0.163** | **0.190** | **5.91** | **1.90** | **3.12** | **0.163** | **0.156** | **5.83** | **2.04** | **2.86** | **0.168** | **0.181** | **5.69** | **1.91** | **2.98** | **0.172** | **0.171** | **5.56** | **1.83** | **3.04** | **0.174** | **0.156** |
|  | **3.29** | **1.09** | **3.03** | **0.238** | **0.081** | **5.11** | **1.57** | **3.24** | **0.190** | **0.121** | **4.98** | **1.55** | **3.22** | **0.186** | **0.127** | **4.80** | **1.56** | **3.08** | **0.201** | **0.125** | **4.69** | **1.46** | **3.22** | **0.201** | **0.113** | **4.56** | **1.46** | **3.12** | **0.206** | **0.115** | **4.63** | **1.46** | **3.18** | **0.207** | **0.102** | **4.72** | **1.48** | **3.20** | **0.206** | **0.113** |
|  | **3.38** | **1.00** | **3.36** | **0.224** | **0.075** | **5.06** | **1.68** | **3.02** | **0.218** | **0.106** | **5.43** | **1.76** | **3.08** | **0.213** | **0.113** | **5.43** | **1.82** | **2.98** | **0.204** | **0.129** | **5.38** | **1.78** | **3.02** | **0.200** | **0.133** | **5.48** | **1.75** | **3.14** | **0.190** | **0.125** | **5.50** | **1.73** | **3.18** | **0.199** | **0.123** | **5.26** | **1.57** | **3.36** | **0.194** | **0.106** |
|  | **4.43** | **1.17** | **3.77** | **0.183** | **0.075** | **5.77** | **1.53** | **3.78** | **0.158** | **0.106** | **6.15** | **1.71** | **3.61** | **0.153** | **0.123** | **6.28** | **1.70** | **3.69** | **0.154** | **0.110** | **6.52** | **1.66** | **3.93** | **0.146** | **0.108** | **6.67** | **1.78** | **3.75** | **0.147** | **0.119** | **6.52** | **1.94** | **3.36** | **0.164** | **0.133** | **6.38** | **1.81** | **3.53** | **0.157** | **0.125** |
|  | **3.61** | **0.95** | **3.81** | **0.175** | **0.079** | **6.49** | **1.59** | **4.07** | **0.139** | **0.104** | **7.19** | **1.71** | **4.21** | **0.122** | **0.113** | **7.84** | **1.81** | **4.32** | **0.117** | **0.115** | **7.89** | **1.83** | **4.32** | **0.117** | **0.115** | **8.39** | **1.94** | **4.32** | **0.117** | **0.117** | **8.22** | **1.93** | **4.25** | **0.118** | **0.117** | **8.39** | **2.06** | **4.07** | **0.125** | **0.121** |
|  | **3.69** | **1.12** | **3.29** | **0.188** | **0.110** | **5.77** | **1.71** | **3.38** | **0.156** | **0.135** | **6.25** | **1.94** | **3.22** | **0.164** | **0.150** | **6.49** | **2.03** | **3.20** | **0.169** | **0.142** | **6.25** | **1.99** | **3.14** | **0.171** | **0.150** | **6.28** | **1.96** | **3.20** | **0.169** | **0.140** | **6.45** | **1.95** | **3.31** | **0.161** | **0.142** | **6.59** | **2.10** | **3.14** | **0.163** | **0.156** |
|  | **3.57** | **0.97** | **3.69** | **0.152** | **0.117** | **5.83** | **1.53** | **3.81** | **0.139** | **0.125** | **6.74** | **1.97** | **3.43** | **0.146** | **0.146** | **6.86** | **2.06** | **3.33** | **0.140** | **0.160** | **6.82** | **2.13** | **3.20** | **0.146** | **0.169** | **6.49** | **2.00** | **3.24** | **0.149** | **0.160** | **6.19** | **1.93** | **3.20** | **0.156** | **0.160** | **6.12** | **1.84** | **3.33** | **0.154** | **0.148** |
|  | **3.72** | **1.00** | **3.73** | **0.185** | **0.075** | **5.97** | **1.47** | **4.07** | **0.146** | **0.096** | **6.70** | **1.72** | **3.90** | **0.140** | **0.117** | **6.90** | **1.81** | **3.81** | **0.138** | **0.125** | **6.94** | **1.85** | **3.75** | **0.140** | **0.127** | **7.19** | **2.01** | **3.58** | **0.144** | **0.133** | **6.90** | **1.95** | **3.53** | **0.150** | **0.133** | **7.06** | **2.01** | **3.50** | **0.146** | **0.142** |
|  | **3.97** | **0.95** | **4.19** | **0.157** | **0.079** | **5.63** | **1.31** | **4.29** | **0.140** | **0.092** | **6.15** | **1.45** | **4.25** | **0.139** | **0.098** | **6.49** | **1.57** | **4.14** | **0.133** | **0.113** | **6.45** | **1.52** | **4.25** | **0.133** | **0.104** | **6.67** | **1.60** | **4.17** | **0.143** | **0.102** | **6.59** | **1.61** | **4.10** | **0.143** | **0.098** | **6.78** | **1.65** | **4.10** | **0.144** | **0.104** |
|  | **3.93** | **1.11** | **3.53** | **0.174** | **0.107** | **5.83** | **1.66** | **3.50** | **0.164** | **0.125** | **6.19** | **1.83** | **3.38** | **0.163** | **0.131** | **6.35** | **1.88** | **3.38** | **0.163** | **0.133** | **6.56** | **1.97** | **3.33** | **0.172** | **0.133** | **6.35** | **1.92** | **3.31** | **0.174** | **0.129** | **6.35** | **2.05** | **3.10** | **0.175** | **0.146** | **5.94** | **1.83** | **3.24** | **0.181** | **0.129** |
|  | **3.90** | **1.15** | **3.38** | **0.185** | **0.107** | **5.88** | **1.64** | **3.58** | **0.158** | **0.119** | **6.49** | **1.81** | **3.58** | **0.157** | **0.127** | **6.82** | **1.83** | **3.72** | **0.149** | **0.123** | **6.56** | **2.05** | **3.20** | **0.157** | **0.158** | **6.49** | **2.05** | **3.16** | **0.156** | **0.158** | **6.25** | **1.88** | **3.33** | **0.157** | **0.144** | **6.25** | **1.97** | **3.18** | **0.169** | **0.152** |
|  | **3.80** | **1.13** | **3.35** | **0.204** | **0.085** | **5.43** | **1.46** | **3.72** | **0.197** | **0.071** | **5.58** | **1.66** | **3.36** | **0.190** | **0.102** | **6.03** | **1.71** | **3.53** | **0.183** | **0.098** | **6.12** | **1.71** | **3.58** | **0.183** | **0.096** | **6.19** | **1.75** | **3.53** | **0.182** | **0.104** | **6.28** | **1.75** | **3.58** | **0.168** | **0.110** | **6.12** | **1.71** | **3.58** | **0.174** | **0.100** |
|  | **4.05** | **1.04** | **3.91** | **0.154** | **0.096** | **6.28** | **1.49** | **4.21** | **0.122** | **0.110** | **6.86** | **1.71** | **4.00** | **0.114** | **0.135** | **7.19** | **1.72** | **4.17** | **0.114** | **0.127** | **7.45** | **1.79** | **4.17** | **0.115** | **0.125** | **7.55** | **1.86** | **4.07** | **0.110** | **0.138** | **7.74** | **1.92** | **4.03** | **0.115** | **0.135** | **7.45** | **1.86** | **4.00** | **0.118** | **0.133** |
|  | **3.31** | **1.19** | **2.77** | **0.214** | **0.129** | **5.45** | **1.69** | **3.22** | **0.165** | **0.146** | **6.03** | **2.06** | **2.93** | **0.163** | **0.179** | **6.28** | **2.00** | **3.14** | **0.165** | **0.152** | **6.42** | **1.98** | **3.24** | **0.154** | **0.156** | **6.45** | **2.15** | **3.00** | **0.158** | **0.179** | **6.56** | **2.16** | **3.04** | **0.164** | **0.165** | **6.38** | **2.15** | **2.96** | **0.169** | **0.167** |
|  | **3.57** | **1.13** | **3.16** | **0.207** | **0.097** | **5.36** | **1.43** | **3.75** | **0.160** | **0.104** | **6.03** | **1.62** | **3.72** | **0.151** | **0.117** | **6.35** | **1.68** | **3.78** | **0.146** | **0.121** | **6.67** | **1.79** | **3.72** | **0.143** | **0.123** | **6.59** | **1.76** | **3.75** | **0.142** | **0.125** | **6.82** | **1.99** | **3.43** | **0.143** | **0.144** | **6.32** | **1.63** | **3.87** | **0.147** | **0.117** |
| **Fast-rhythm** | **S - 5m** |  |  |  |  | **5m - 10m** |  |  |  |  | **10m - 15m** |  |  |  |  | **15m - 20m** |  |  |  |  | **20m - 25m** |  |  |  |  | **25m - 30m** |  |  |  |  | **30m - 35m** |  |  |  |  | **35m - 40m** |  |  |  |  |
|  | **Sprint velocity** | **Step length** | **Step rate** | **Stance time** | **Flight time** | **Sprint velocity** | **Step length** | **Step rate** | **Stance time** | **Flight time** | **Sprint velocity** | **Step length** | **Step rate** | **Stance time** | **Flight time** | **Sprint velocity** | **Step length** | **Step rate** | **Stance time** | **Flight time** | **Sprint velocity** | **Step length** | **Step rate** | **Stance time** | **Flight time** | **Sprint velocity** | **Step length** | **Step rate** | **Stance time** | **Flight time** | **Sprint velocity** | **Step length** | **Step rate** | **Stance time** | **Flight time** | **Sprint velocity** | **Step length** | **Step rate** | **Stance time** | **Flight time** |
|  | **4.27** | **1.03** | **4.14** | **0.161** | **0.081** | **6.22** | **1.49** | **4.17** | **0.139** | **0.098** | **6.86** | **1.57** | **4.36** | **0.126** | **0.106** | **7.55** | **1.79** | **4.21** | **0.124** | **0.113** | **7.74** | **1.79** | **4.32** | **0.122** | **0.110** | **7.84** | **1.80** | **4.36** | **0.122** | **0.108** | **7.95** | **1.85** | **4.29** | **0.121** | **0.115** | **7.84** | **1.76** | **4.44** | **0.122** | **0.104** |
|  | **3.96** | **0.90** | **4.42** | **0.160** | **0.063** | **5.88** | **1.20** | **4.90** | **0.131** | **0.075** | **6.52** | **1.40** | **4.66** | **0.124** | **0.092** | **6.78** | **1.54** | **4.40** | **0.132** | **0.096** | **6.86** | **1.54** | **4.44** | **0.128** | **0.098** | **6.98** | **1.58** | **4.40** | **0.132** | **0.094** | **7.02** | **1.52** | **4.62** | **0.126** | **0.092** | **6.90** | **1.52** | **4.53** | **0.125** | **0.094** |
|  | **4.51** | **1.11** | **4.07** | **0.166** | **0.072** | **5.80** | **1.33** | **4.36** | **0.129** | **0.100** | **6.52** | **1.52** | **4.29** | **0.129** | **0.102** | **6.90** | **1.65** | **4.17** | **0.125** | **0.113** | **7.14** | **1.71** | **4.17** | **0.126** | **0.115** | **7.27** | **1.74** | **4.17** | **0.125** | **0.115** | **7.36** | **1.79** | **4.10** | **0.128** | **0.117** | **7.41** | **1.85** | **4.00** | **0.122** | **0.127** |
|  | **4.17** | **1.07** | **3.89** | **0.144** | **0.108** | **6.06** | **1.44** | **4.21** | **0.126** | **0.110** | **6.67** | **1.47** | **4.53** | **0.121** | **0.100** | **7.14** | **1.61** | **4.44** | **0.122** | **0.102** | **7.27** | **1.71** | **4.25** | **0.121** | **0.117** | **7.32** | **1.78** | **4.10** | **0.119** | **0.121** | **7.32** | **1.80** | **4.07** | **0.124** | **0.125** | **7.19** | **1.83** | **3.93** | **0.126** | **0.131** |
|  | **3.93** | **0.93** | **4.24** | **0.159** | **0.074** | **5.63** | **1.57** | **3.58** | **0.128** | **0.129** | **6.32** | **1.39** | **4.53** | **0.128** | **0.094** | **6.67** | **1.54** | **4.32** | **0.124** | **0.106** | **6.82** | **1.56** | **4.36** | **0.128** | **0.102** | **6.82** | **1.66** | **4.10** | **0.131** | **0.113** | **6.86** | **1.61** | **4.25** | **0.126** | **0.110** | **6.74** | **1.63** | **4.14** | **0.133** | **0.110** |
|  | **3.95** | **1.09** | **3.62** | **0.190** | **0.079** | **5.36** | **1.37** | **3.90** | **0.157** | **0.102** | **5.94** | **1.52** | **3.90** | **0.154** | **0.106** | **6.19** | **1.57** | **3.93** | **0.154** | **0.102** | **6.28** | **1.65** | **3.81** | **0.147** | **0.115** | **6.35** | **1.67** | **3.81** | **0.153** | **0.110** | **6.22** | **1.68** | **3.69** | **0.153** | **0.117** | **6.00** | **1.65** | **3.64** | **0.157** | **0.119** |
|  | **3.47** | **0.85** | **4.07** | **0.205** | **0.038** | **5.02** | **1.20** | **4.17** | **0.188** | **0.056** | **5.11** | **1.24** | **4.10** | **0.189** | **0.056** | **5.33** | **1.36** | **3.93** | **0.183** | **0.071** | **5.41** | **1.40** | **3.87** | **0.193** | **0.069** | **5.43** | **1.39** | **3.90** | **0.183** | **0.069** | **5.45** | **1.38** | **3.97** | **0.181** | **0.071** | **5.22** | **1.34** | **3.90** | **0.190** | **0.063** |
|  | **3.97** | **1.05** | **3.77** | **0.174** | **0.083** | **5.80** | **1.49** | **3.90** | **0.142** | **0.115** | **6.38** | **1.61** | **3.97** | **0.135** | **0.117** | **6.78** | **1.74** | **3.90** | **0.139** | **0.121** | **6.74** | **1.83** | **3.69** | **0.136** | **0.135** | **6.82** | **1.85** | **3.69** | **0.144** | **0.131** | **6.94** | **1.94** | **3.58** | **0.142** | **0.131** | **6.74** | **1.85** | **3.64** | **0.144** | **0.131** |
|  | **3.90** | **1.06** | **3.69** | **0.220** | **0.044** | **5.74** | **1.44** | **4.00** | **0.174** | **0.075** | **6.22** | **1.53** | **4.07** | **0.161** | **0.085** | **6.52** | **1.60** | **4.07** | **0.156** | **0.088** | **6.67** | **1.56** | **4.29** | **0.156** | **0.079** | **6.74** | **1.67** | **4.03** | **0.151** | **0.094** | **6.82** | **1.66** | **4.10** | **0.150** | **0.098** | **6.67** | **1.83** | **3.64** | **0.164** | **0.113** |
|  | **3.49** | **0.95** | **3.67** | **0.190** | **0.071** | **5.71** | **1.39** | **4.10** | **0.154** | **0.090** | **6.22** | **1.54** | **4.03** | **0.154** | **0.092** | **6.28** | **1.61** | **3.90** | **0.160** | **0.094** | **6.25** | **1.71** | **3.66** | **0.172** | **0.094** | **6.28** | **1.68** | **3.75** | **0.156** | **0.106** | **6.35** | **1.67** | **3.81** | **0.167** | **0.094** | **6.15** | **1.64** | **3.75** | **0.167** | **0.094** |
|  | **4.35** | **1.06** | **4.11** | **0.169** | **0.068** | **6.25** | **1.43** | **4.36** | **0.149** | **0.081** | **6.74** | **1.54** | **4.36** | **0.138** | **0.092** | **7.06** | **1.60** | **4.40** | **0.139** | **0.090** | **7.10** | **1.63** | **4.36** | **0.138** | **0.092** | **7.23** | **1.73** | **4.17** | **0.135** | **0.102** | **7.23** | **1.70** | **4.25** | **0.140** | **0.092** | **7.10** | **1.69** | **4.21** | **0.140** | **0.100** |
|  | **3.97** | **0.93** | **4.26** | **0.165** | **0.061** | **6.63** | **1.46** | **4.53** | **0.124** | **0.094** | **7.69** | **1.67** | **4.62** | **0.117** | **0.102** | **8.33** | **1.81** | **4.62** | **0.115** | **0.100** | **8.39** | **1.78** | **4.71** | **0.111** | **0.102** | **8.76** | **1.92** | **4.57** | **0.111** | **0.108** | **8.76** | **1.95** | **4.49** | **0.115** | **0.106** | **8.96** | **2.03** | **4.40** | **0.111** | **0.115** |
|  | **4.07** | **1.14** | **3.58** | **0.188** | **0.083** | **6.00** | **1.53** | **3.93** | **0.144** | **0.106** | **6.67** | **1.68** | **3.97** | **0.142** | **0.110** | **7.14** | **1.80** | **3.97** | **0.139** | **0.110** | **7.27** | **1.82** | **4.00** | **0.139** | **0.110** | **7.55** | **1.90** | **3.97** | **0.136** | **0.115** | **7.55** | **1.90** | **3.97** | **0.136** | **0.117** | **7.74** | **1.92** | **4.03** | **0.139** | **0.108** |
|  | **3.82** | **0.98** | **3.91** | **0.163** | **0.088** | **6.35** | **1.42** | **4.49** | **0.122** | **0.098** | **7.45** | **1.82** | **4.10** | **0.122** | **0.117** | **8.05** | **1.91** | **4.21** | **0.119** | **0.119** | **8.16** | **2.01** | **4.07** | **0.124** | **0.123** | **7.95** | **1.92** | **4.14** | **0.122** | **0.123** | **7.64** | **1.99** | **3.84** | **0.126** | **0.135** | **7.50** | **1.66** | **4.53** | **0.124** | **0.100** |
|  | **3.99** | **0.94** | **4.26** | **0.157** | **0.074** | **6.52** | **1.44** | **4.53** | **0.138** | **0.083** | **7.32** | **1.65** | **4.44** | **0.124** | **0.102** | **7.89** | **1.79** | **4.40** | **0.125** | **0.102** | **8.05** | **1.78** | **4.53** | **0.126** | **0.096** | **8.33** | **1.86** | **4.49** | **0.131** | **0.094** | **8.11** | **1.98** | **4.10** | **0.142** | **0.102** | **8.05** | **2.03** | **3.97** | **0.147** | **0.106** |
|  | **4.03** | **0.91** | **4.44** | **0.151** | **0.069** | **5.80** | **1.29** | **4.49** | **0.140** | **0.083** | **6.19** | **1.40** | **4.40** | **0.142** | **0.081** | **6.49** | **1.45** | **4.49** | **0.136** | **0.090** | **6.45** | **1.57** | **4.10** | **0.138** | **0.104** | **6.56** | **1.53** | **4.29** | **0.138** | **0.098** | **6.56** | **1.56** | **4.21** | **0.142** | **0.098** | **6.63** | **1.63** | **4.07** | **0.151** | **0.098** |
|  | **3.85** | **0.99** | **3.87** | **0.182** | **0.071** | **6.09** | **1.46** | **4.17** | **0.153** | **0.085** | **6.63** | **1.59** | **4.17** | **0.147** | **0.094** | **6.94** | **1.65** | **4.21** | **0.153** | **0.085** | **7.02** | **1.84** | **3.81** | **0.163** | **0.104** | **6.94** | **1.73** | **4.00** | **0.157** | **0.092** | **7.14** | **1.88** | **3.81** | **0.157** | **0.102** | **6.90** | **1.72** | **4.00** | **0.169** | **0.088** |
|  | **4.17** | **1.08** | **3.85** | **0.175** | **0.079** | **5.91** | **1.50** | **3.93** | **0.147** | **0.106** | **6.63** | **1.74** | **3.81** | **0.147** | **0.117** | **6.78** | **1.72** | **3.93** | **0.144** | **0.110** | **6.78** | **1.72** | **3.93** | **0.149** | **0.104** | **6.82** | **1.73** | **3.93** | **0.149** | **0.106** | **6.67** | **1.74** | **3.84** | **0.146** | **0.115** | **6.78** | **1.77** | **3.84** | **0.146** | **0.113** |
|  | **3.72** | **1.01** | **3.67** | **0.218** | **0.044** | **5.91** | **1.43** | **4.14** | **0.185** | **0.054** | **6.38** | **1.54** | **4.14** | **0.182** | **0.063** | **6.56** | **1.64** | **4.00** | **0.176** | **0.071** | **6.86** | **1.66** | **4.14** | **0.167** | **0.075** | **6.67** | **1.67** | **4.00** | **0.178** | **0.075** | **6.78** | **1.77** | **3.84** | **0.169** | **0.088** | **6.67** | **1.68** | **3.97** | **0.167** | **0.085** |
|  | **3.91** | **0.96** | **4.07** | **0.146** | **0.096** | **6.25** | **1.43** | **4.36** | **0.119** | **0.108** | **6.94** | **1.56** | **4.44** | **0.113** | **0.113** | **7.36** | **1.72** | **4.29** | **0.110** | **0.123** | **7.55** | **1.81** | **4.17** | **0.108** | **0.133** | **7.64** | **1.88** | **4.07** | **0.114** | **0.131** | **7.69** | **1.92** | **4.00** | **0.110** | **0.140** | **7.64** | **1.85** | **4.14** | **0.111** | **0.131** |
|  | **4.05** | **1.19** | **3.41** | **0.184** | **0.097** | **5.97** | **1.57** | **3.81** | **0.147** | **0.117** | **6.56** | **1.73** | **3.78** | **0.142** | **0.121** | **7.14** | **1.86** | **3.84** | **0.135** | **0.125** | **7.55** | **1.98** | **3.81** | **0.138** | **0.125** | **7.41** | **1.94** | **3.81** | **0.144** | **0.119** | **7.45** | **1.93** | **3.87** | **0.143** | **0.115** | **7.41** | **1.99** | **3.72** | **0.143** | **0.127** |
|  | **4.07** | **1.01** | **4.04** | **0.164** | **0.075** | **6.59** | **1.41** | **4.66** | **0.135** | **0.081** | **7.23** | **1.48** | **4.90** | **0.122** | **0.083** | **7.84** | **1.68** | **4.66** | **0.121** | **0.092** | **8.11** | **1.76** | **4.62** | **0.122** | **0.096** | **8.00** | **1.78** | **4.49** | **0.117** | **0.104** | **8.16** | **1.80** | **4.53** | **0.118** | **0.102** | **8.11** | **1.72** | **4.71** | **0.122** | **0.094** |
