# Supplementary material for: Effects of rhythmic auditory guide on sprint running
Source: PLoS One. 2025 Mar 21;20(3):e0319738. doi: 10.1371/journal.pone.0319738 (PMC11927899; doi:10.1371/journal.pone.0319738)
Supplement: S2 Table — (DOCX) [file pone.0319738.s002.docx]

**S2 Table. The relative phase between in-phase and antiphase percentages in Slow and Fast-rhythm tasks for each participant**

| **Slow-rhythm** | | **Fast-rhythm** | |
| --- | --- | --- | --- |
| **Relative phase (°)** | | **Relative phase (°)** | |
| **In-phase** | **Antiphase** | **In-phase** | **Antiphase** |
| **0.68** | **0.32** | **0.96** | **0.04** |
| **0.72** | **0.28** | **0.11** | **0.89** |
| **0.35** | **0.65** | **1.00** | **0.00** |
| **0.78** | **0.22** | **0.54** | **0.46** |
| **1.00** | **0.00** | **0.42** | **0.58** |
| **0.76** | **0.24** | **0.36** | **0.64** |
| **0.80** | **0.20** | **0.07** | **0.93** |
| **0.91** | **0.09** | **0.48** | **0.52** |
| **0.48** | **0.52** | **0.40** | **0.60** |
| **0.96** | **0.04** | **0.73** | **0.27** |
| **0.57** | **0.43** | **0.52** | **0.48** |
| **0.41** | **0.59** | **0.00** | **1.00** |
| **0.82** | **0.18** | **0.04** | **0.96** |
| **0.35** | **0.65** | **1.00** | **0.00** |
| **0.72** | **0.28** | **0.54** | **0.46** |
| **0.56** | **0.44** | **0.36** | **0.64** |
| **0.77** | **0.23** | **0.72** | **0.28** |
| **0.32** | **0.68** | **0.48** | **0.52** |
| **0.64** | **0.36** | **0.38** | **0.62** |
| **0.41** | **0.59** | **0.84** | **0.16** |
| **0.57** | **0.43** | **0.45** | **0.55** |
| **0.46** | **0.54** | **0.58** | **0.42** |
